# Decoding the Divergent Subcellular Location of Two Highly Similar Paralogous LEA Proteins

**DOI:** 10.3390/ijms19061620

**Published:** 2018-05-31

**Authors:** Marie-Hélène Avelange-Macherel, Adrien Candat, Martine Neveu, Dimitri Tolleter, David Macherel

**Affiliations:** IRHS, Agrocampus-Ouest, INRA, Université d’Angers, SFR 4207 Quasav, 42 rue George Morel, 49071 Beaucouzé, France; marie-helene.macherel@agrocampus-ouest.fr (M.-H.A.-M.); adrienpel@gmail.com (A.C.); martine.neveu@inra.fr (M.N.); dimitri.tolleter@univ-angers.fr (D.T.)

**Keywords:** late embryogenesis abundant protein, mitochondrion, mitochondrial import, gene duplication, paralog

## Abstract

Many mitochondrial proteins are synthesized as precursors in the cytosol with an N-terminal mitochondrial targeting sequence (MTS) which is cleaved off upon import. Although much is known about import mechanisms and MTS structural features, the variability of MTS still hampers robust sub-cellular software predictions. Here, we took advantage of two paralogous late embryogenesis abundant proteins (LEA) from Arabidopsis with different subcellular locations to investigate structural determinants of mitochondrial import and gain insight into the evolution of the *LEA* genes. LEA38 and LEA2 are short proteins of the LEA_3 family, which are very similar along their whole sequence, but LEA38 is targeted to mitochondria while LEA2 is cytosolic. Differences in the N-terminal protein sequences were used to generate a series of mutated LEA2 which were expressed as GFP-fusion proteins in leaf protoplasts. By combining three types of mutation (substitution, charge inversion, and segment replacement), we were able to redirect the mutated LEA2 to mitochondria. Analysis of the effect of the mutations and determination of the LEA38 MTS cleavage site highlighted important structural features within and beyond the MTS. Overall, these results provide an explanation for the likely loss of mitochondrial location after duplication of the ancestral gene.

## 1. Introduction

Mitochondria are key organelles of eukaryotic cells involved in energy production, metabolism, and signaling. Since only a few dozen proteins are encoded in the mitochondrial genome, several thousand proteins need to be imported into the organelle [1,2]. Mitochondrial targeted proteins are first synthesized in the cytosol as precursors and often display a mitochondrial targeting sequence (MTS) in their N-terminus. However, precursors for inner membrane proteins do not exhibit an MTS but internal targeting signals [3,4]. Precursor proteins are translocated as unfolded polypeptides across mitochondrial membranes via the transporter of the outer membrane complex (TOM) and the transporter of the inner membrane complex (TIM) [3,4]. According to the binding chain hypothesis [5], import proceeds through successive MTS binding to higher affinity sites, until the protein is trapped in the mitochondrial matrix by mitochondrial chaperones. While crossing the outer membrane does not require energy, an electrochemical proton gradient and ATP are needed for inner membrane pre-protein translocation [6]. MTS are generally cleaved off during the passage through mitochondrial membranes, or in the matrix, by the mitochondrial processing peptidase (MPP) or other peptidases, and further degraded by proteases [7]. Although it was generally considered that MTS were systematically cleaved upon import, which happens for the majority of matrix proteins, there is now increasing evidence that possibly half of mitochondrial proteins could be imported without cleavage of the MTS [2]. The overall mechanisms of mitochondrial protein import are similar in animals, yeast, and plants, although plant Tom20 does not appear to be orthologous to the animal and yeast Tom20 proteins [8]. Besides, the plant MPP peptidase is not localized in the matrix but is part of the cytochrome bc1 complex of the respiratory chain [9]. Protein import in plastids proceeds in a similar way as in mitochondria, but with different translocators (for review, [10]), and it is therefore not surprising that mitochondrial and plastidial pre-sequences share some common features and are not easily distinguished, although mitochondrial MTS generally contain more arginine [11]. Moreover, there is an increasing number of proteins which appear dually targeted to both organelles [12]. MTS share common properties because they carry essential information for proper targeting of several hundred mitochondrial proteins. However, they are still insufficiently characterized, partly because of their structural diversity, but also because of their similarity to plastid transit peptides. MTS length was found to be highly variable: 6–94 amino acids in yeast, 19–109 amino acids in Arabidopsis, and 1–122 in *Oryza sativa* [13]. Regarding amino acid composition, MTS contain a high proportion of positively charged, hydroxylated, and hydrophobic residues, but few acidic amino acids [14], and plant pre-sequences are noticeably enriched in serine [15].

MTS have the propensity to fold into an amphiphilic alpha-helix (with opposing positively charged and hydrophobic faces), a structure that would favor the interactions with TOM components and was considered to be necessary and sufficient for import [14,16,17]. Interestingly, in spite of the functional similarity between mitochondrial and plastid import systems, chloroplast transit peptides are usually unstructured although they could also form a helix upon contact with membranes [18]. No clear consensus sequence has been found for MTS, but loosely conserved motifs with Arg at the -2, -3, or -10 position from the processing site have been identified: -2R motif {R-X**↓**X}, -3R motif {R-X-(F/Y/L)**↓**(A/S)-X}, and -10R motif {R-X-(F/L/I)-X_2_-(T/S/G)-X_4_**↓**X} [7,14,19]. The Arg residue was experimentally shown to be important for cleavage processing [20]. Indeed, MPP recognises basic amino-acids and cleaves the sequence motif {(R/K)-Xn-R-X**↓**Φ-Ψ-Ψ} (with Φ and Ψ hydrophobic and hydrophilic residues, respectively) more efficiently [21]. However, pre-sequences without any conserved arginine close to the cleavage site (no-R motif) were also reported [22]. Studies in plants revealed a conserved motif {(F/Y)**↓**(S/A)} for the no-R group of proteins [13]. The -2R, -3R, and no-R motifs were found in pre-sequences of all organisms, whereas the -10R motif was not found in plants [13,22]. MTS features around the cleavage site have been thoroughly investigated in Arabidopsis and rice [13]. In these species, the -3R motif occurs more frequently in Arabidopsis MTS with Phe in the -1 position with respect to cleavage, or Phe, Tyr, and Leu in the case of rice MTS. Less flexibility was observed for plant MTS compared to non-plant MTS. Indeed, the presence of plastids may require a higher specificity of plant MTS in order to prevent mistargeting [23].

A number of publicly available online computer programs have been developed to predict mitochondrial targeted proteins, but they display poor consensus when comparing their predictions for a large number of protein sequences [24]. The programs are trained by using only a small number of proteins and their often ambiguous conclusions are due, in part, to the high diversity of MTS. A deeper understanding of the functional features of MTS, as well as larger bodies of experimental MTS data, are therefore needed to identify the key determinants for mitochondrial targeting and improve predictors.

In *Arabidopsis thaliana* Col-0, the subcellular locations of 51 proteins belonging to the Late Embryogenesis Abundant (LEA) protein family were determined experimentally using translational fusions with fluorescent proteins [25]. LEA proteins are characterized by a low sequence complexity, repeated motifs, and high hydrophilicity, and are often intrinsically disordered [26]. Among the 51 LEA proteins, five were found to be targeted to mitochondria. Two of them (LEA42, At4g15910; LEA48, At5g44310) belong to the PFAM family LEA_4, whose members were found to be distributed in many cellular compartments, and are expected to protect various cellular membranes during desiccation. LEA42 and LEA48 were dually targeted to mitochondria and chloroplasts [25]. They are expected to play a similar role to their orthologous pea protein LEAM in protecting the inner mitochondrial membrane during desiccation [27,28,29]. Three other LEA proteins (LEA37, At3g53770; LEA38, At4g02380; LEA41, At4g15910) were found to be exclusively mitochondrial. They belong to the PFAM LEA_3 family, which encompasses four members in Arabidopsis. Interestingly, this fourth member (LEA2, At1g02820), which is a paralogous protein to LEA38, was localized to the cytosol [25]. In contrast with proteins from the LEA_4 family which appears to be involved in seed desiccation tolerance, little is known about the function of proteins from the LEA_3 family [26]. *LEA38* was first identified as a senescence associated gene (*SAG21*) [30], and then as a gene (*AtLEA5*) able to complement an oxidant-stress sensitive yeast mutant [31]. Further work using overexpressor and antisense lines led to the proposal that LEA38 is involved in root development and abiotic stress tolerance [32]. However, to our knowledge, there is still no clue about the molecular function of any protein of the LEA_3 family. Excitingly, a genome-wide association study (GWAS) aiming to identify *Arabidopsis thaliana* loci involved in local geographic adaptation highlighted *LEA38* among the four best hits, suggesting a key role for the LEA38 protein in environmental adaptation [33].

Whatever their function, LEA38 and LEA2 are two paralogous proteins of a small size (around 10 kDa), with highly similar sequences but different subcellular locations [25]. These proteins therefore represent an original model to uncover MTS specific motifs, and to explore how these two paralogous genes evolved to encode proteins with different subcellular targeting. Here, bioinformatics, genetic engineering, and subcellular localization of synthetic proteins were used to identify the key determinants for mitochondrial localization of LEA38, which were likely lost in LEA2 after gene duplication.

## 2. Results

### 2.1. Proteins of the LEA_3 Family Are Expected to Be Mitochondrial

In Arabidopsis, the LEA_3 family comprises four proteins, with three of them (including LEA38) being exclusively mitochondrial, while the fourth (LEA2) is cytosolic [25]. This suggests that LEA2, which is paralogous to LEA38, could have lost its mitochondrial localization during evolution. To support this hypothesis, we first examined whether mitochondrial localization could possibly be the rule for LEA_3 proteins. The LEA38 sequence was used as a query in a BLASTP search of the NCBI Reference Sequence protein database. With an E-value cut-off of 10, the analysis yielded 390 hits (370 hits with *e*-value < 0.01). All the sequences were from higher plants, and a search for PFAM matches confirmed that all these sequences belonged to the PFAM family LEA_3 (PF03242). This strongly suggests that this protein family is specific to higher plants. The 390 protein sequences were then subjected to subcellular localization prediction using PProwler, which proved to be the more accurate prediction program for LEA proteins [25].

**Figure 1:**
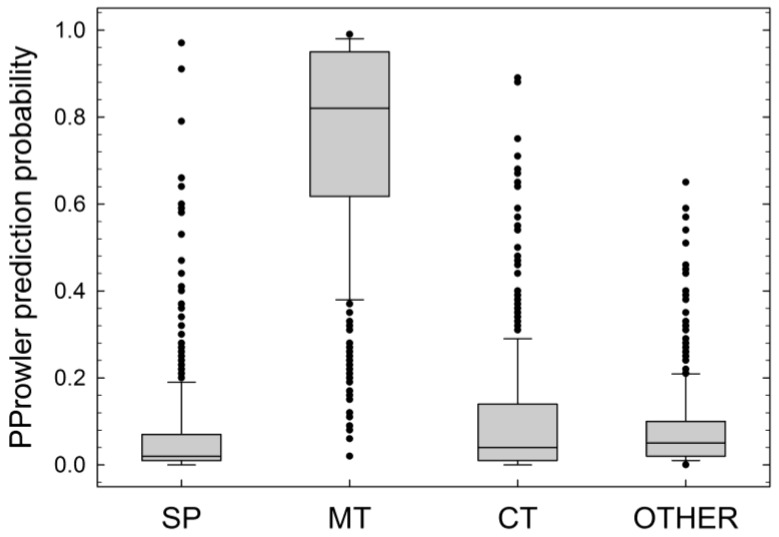
Subcellular prediction for proteins of the LEA_3 family. The graph shows the probability of subcellular targeting for 390 proteins of the LEA_3 family to different compartments (SP, secretory pathway; MT, mitochondria targeting; CT, chloroplast targeting) according to the PProwler software. Peroxisomal targeting probability is not indicated (null for all proteins). The 390 proteins selected are the best matches retrieved using the LEA38 protein sequence as the query in a BLASTP search against the NCBI Reference Sequence protein database, and all belong to the LEA_3 family. The boundary of the box closest to zero indicates the 25th percentile, a line within the box marks the median, and the boundary of the box farthest from zero indicates the 75th percentile. Whiskers (error bars) above and below the box indicate the 90th and 10th percentile, respectively.

The program computes probabilities for localization in the secretory pathway, mitochondrion, chloroplast, peroxisome, and others (nucleus, cytoplasmic, or otherwise). The box plot shown in Figure 1 clearly reveals that the mitochondrion is by far the most probable subcellular destination of LEA_3 proteins. Therefore, it is likely that within the LEA38/LEA2 couple of paralogs, LEA38 has retained its mitochondrial localization, while LEA2 has lost it.

### 2.2. Determinants of LEA38 Mitochondrial Targeting

**Figure 2:**
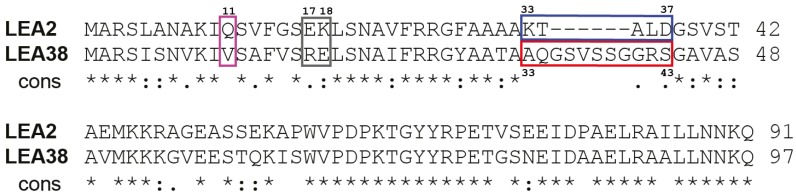
Comparison of LEA2 and LEA38 protein sequences. Amino acid alignment was performed with LALIGN. The degree of similarity between amino acids occupying the same position is indicated by the following symbols: “*” identity; “:” strong similarity; “.” weak similarity. Sequence differences selected to generate mutated LEA2 proteins are indicated by colored boxes.

As shown in Figure 2, LEA2 and LEA38, which share 60.8% identity and 80.4% similarity, as calculated by LALIGN, are very similar proteins, even in their N-terminal part, which is a critical region for organelle targeting. However, four differences are deemed of interest and are highlighted in Figure 2: a change in polarity at position 11 (Gln in LEA2, Val in LEA38), a charge inversion at position 17 and 18 (E17-K18 in LEA2, R17-E18 in LEA38), a stretch of 11 amino acids (^33^AQGSVSSGGRS) specific to LEA38, and a segment of five amino acids (^33^KTALD) specific to LEA2 in the same region.

To assess the importance of these modifications with respect to mitochondrial targeting of LEA38, we generated a series of synthetic genes encoding mutated LEA2 and LEA38 proteins (Table 1).

**Figure 3:**
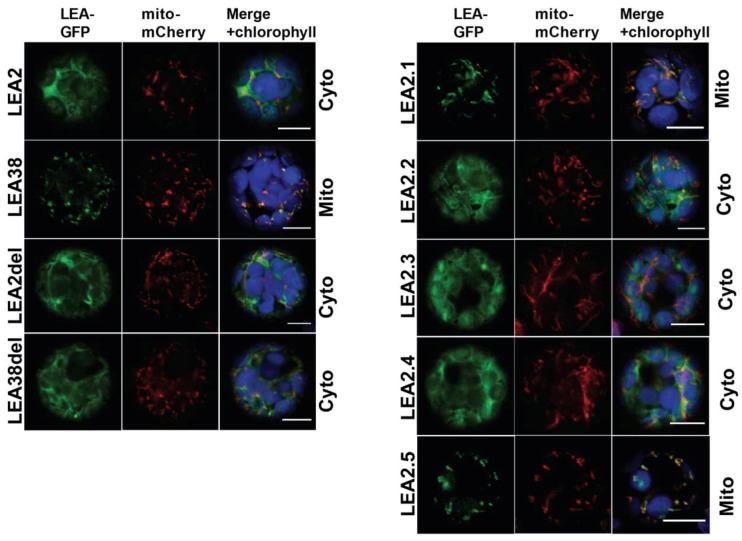
Impact of the mutations on the subcellular localization of LEA2-GFP and LEA38-GFP. The different proteins were expressed in fusion with GFP in Arabidopsis leaf protoplasts, using a line constitutively expressing a mitochondrial mCherry fluorescent protein. Observations were performed with a confocal microscope to visualize fluorescence from GFP, mCherry, and chlorophyll. First column: GFP, green fluorescence; second column: mCherry, red fluorescence; third column: merging of GFP and mCherry signals with chlorophyll autofluorescence in blue. Cyto, cytosolic; Mito, mitochondrial. Bars = 10 µm.

The mutated genes were cloned into an expression vector in order to express the proteins of interest as translational fusions with GFP at their C-terminus. The constructs were used to transiently transform protoplasts from an Arabidopsis line expressing a mitochondrial mCherry marker [25]. LEA2-GFP and LEA38-GFP, used as controls, displayed their expected cytosolic and mitochondrial localizations, respectively (Figure 3).

LEA2del-GFP, in which the ^33^KTALD segment has been deleted, remained cytosolic, indicating that this sequence specific of LEA2 was not preventing mitochondrial targeting, having apparently no detectable effect on subcellular localization (Figure 3). LEA2.1 was constructed by replacing the first 37 amino acids of LEA2 with the first 43 amino acids of LEA38, including its specific ^33^AQGSVSSGGRS segment (Figure 2; Table 1). LEA2.1-GFP systematically accumulated in mitochondria (Figure 3), suggesting that critical information for mitochondrial targeting is indeed enclosed in the first 43 amino acids of LEA38. The LEA38del-GFP construct, in which the specific ^33^AQGSVSSGGRS segment was deleted, was no longer targeted to mitochondria, and remained cytosolic (Figure 3). This motif is therefore essential for mitochondrial targeting of LEA38. To determine if this motif could confer mitochondrial targeting to LEA2, LEA2 was modified to replace its ^33^KTALD segment with the ^33^AQGSVSSGGRS motif of LEA38, yielding the LEA2.2 construct (Figure 2; Table 1). However, the modified LEA2.2-GFP remained localized in the cytosol. Together with the LEA38del-GFP construct, these results indicate that the ^33^AQGSVSSGGRS sequence is essential but not sufficient to confer mitochondrial targeting to LEA38 or LEA2.

To take into account the possible role of other sequence differences between LEA2 and LEA38, three additional constructs were generated from LEA2.2 (Table 1). First, the hydrophilic Gln residue at position 11 of LEA2 was replaced with the hydrophobic Val residue found in LEA38 to yield the LEA2.3 construct (Table 1). Secondly, the two residues E17-K18 in the LEA2 sequence were inversed into K17-E18 to mimic the corresponding charge arrangement found in LEA38 (R17-E18), yielding the construct LEA2.4 (Table 1). Finally, these two mutations were combined, yielding the LEA2.5 construct. When expressed in protoplasts, LEA2.3-GFP and LEA2.4-GFP systematically remained cytosolic (Figure 3). However, in the case of LEA2.5-GFP, which combines all candidate mutations, mitochondrial localization could finally be demonstrated (Figure 3). With other constructs (LEA2del; LEA38del; LEA2.1; LEA2.2; LEA2.3; LEA2.4), all transformed protoplasts showed a reporter GFP with cytosolic localization. However, in the case of LEA2.5-GFP, mitochondrial localization was not observed in all transformed protoplasts. Instead, approximately half of the transformed protoplasts (85/177 protoplasts from seven experiments) showed mitochondrial localization, while the others showed a diffuse cytosolic localization or large aggregates of GFP. We never observed a dual localization of LEA2.5 in mitochondria and cytosol (or aggregates). Although it is difficult to provide a rationale for this heterogeneity, it could be due to the fact that more mutations in the N-terminal sequence could be required to fully restore the efficiency of import, especially in the protoplast assay in which transgene expression is very strong. Nevertheless, the results indicate that while the specific KTALD segment of LEA2 is not involved in subcellular localization of the protein, the addition of the LEA38 specific motif AQGSVSSGGRS, the replacement of Gln11 with Val, and the inversion of E17-K18 are sufficient altogether to target LEA2 to the mitochondrial compartment. It should be noted that none of the synthetic proteins were found to be targeted to the chloroplast, although common features exist for protein import in chloroplasts and mitochondria [18].

**Figure 4:**
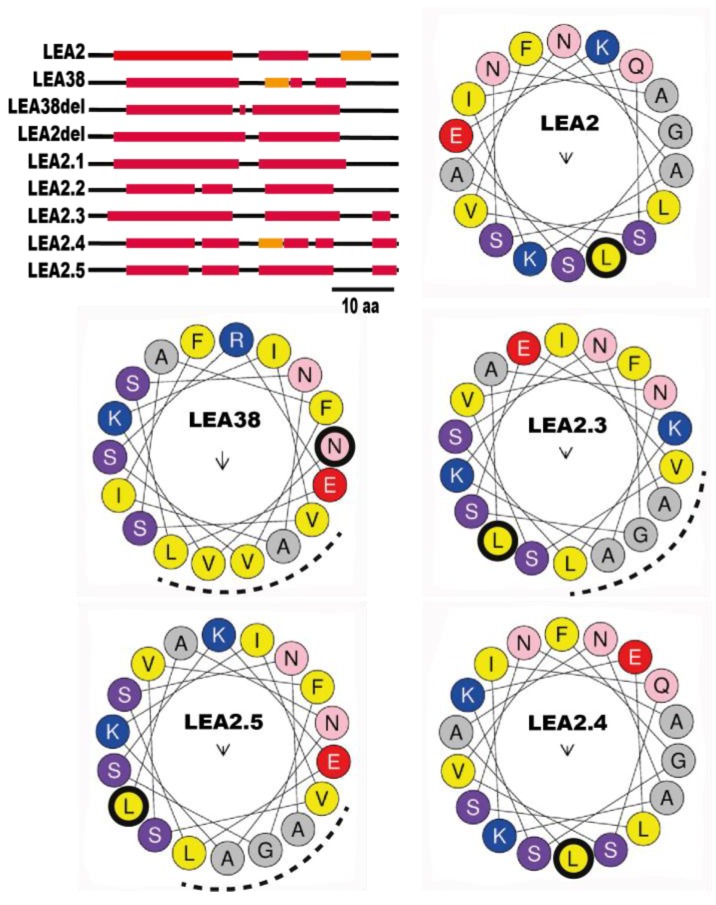
Secondary structure predictions for LEA2, LEA38, and mutated proteins. Secondary structure predictions for the first 50 amino acids (top left part of the figure) were performed using JPRED4 software. Red box: alpha-helix: orange box: extended strand; black line: random coil. Helical projections of α-helices were obtained using 18 amino-acids at the beginning of the first helix in the indicated proteins. The helical projections of LEA38, LEA38del, and LEA2.1, which share the same sequence in the analyzed region, are identical, as well as those of LEA2, LEA2del, and LEA2.2. The first residue is circled in black. Positively charged residues (K, R) are color coded in blue, while negatively charged residues (D, E) are in red. Non-polar residues are shown in yellow or grey and others in purple, light blue, or light pink colors. The arrow of variable size in the center of the helix shows the weight and orientation of the hydrophobic moment, and the dotted line identifies the hydrophobic face.

Since MTS are expected to adopt an amphiphilic helix conformation, the secondary structures of LEA2, LEA38, and mutated proteins were investigated. As shown in Figure 4, all native and mutant proteins were predicted to harbor alpha helix domains in their N-terminal region, irrespective of their subcellular localization. Both LEA2 and LEA38 sequences display two alpha-helices; the first from residues 5–23 and 7–24, respectively, and a second from residues 28–35 and 33–41, respectively, which is preceded by a short extended strand sequence of four residues in LEA38. The five and eleven residue long segments specific to LEA2 or LEA38 are located in this second alpha-helix region. In LEA2del, deletion of the five residues generated a longer helix, and the protein remained cytosolic. In LEA38del, for which mitochondrial localization was abolished, the deletion of the eleven residues removed the small extended strand found in LEA38, yielding a longer helix (Figure 4). This suggests that the extended strand-helical conformation in this region could be important for mitochondrial localization, but the hypothesis can be dismissed because this secondary structure is detected in LEA2.4 (cytosolic) but not in LEA2.5 (mitochondrial). Since the other mutations introduced to redirect LEA2 to mitochondria were localized in the first alpha-helix, helical projections with the Heliquest program were performed to address the structural impact of mutations (Figure 4). The projection of residues 7–24 of LEA38 revealed an amphiphilic helix, with two positively charged residues in the hydrophilic face (VAVVL) and a negatively charged residue (Glu) at the border between the hydrophobic and hydrophilic faces. In LEA2, the projection of residues 5–23 did not reveal a clear amphiphilic face, in spite of the grouping of four residues (AGAL). As found for the corresponding helix in LEA38, three charged residues are present but with a different disposition (Figure 4). The negatively charged residue (Glu) was located at the opposite end of the AGAL region, and the positively charged residues were facing each other, at 90° of the Glu. The Q11V mutation in LEA2.3 restored an amphiphilic alpha-helix with a hydrophobic face (VAGAL), but with a disposition of charged residues similar to LEA2 (Figure 4). The charge inversion (EK/KE) in LEA2.4 restored a disposition of the three charged residues very similar to that in LEA38, but like in LEA2, a hydrophobic face could not be identified (Figure 4). In LEA2.5, in which these two latter mutations were combined, the alpha-helix shows a clear amphiphilic profile with a charged residue distribution almost identical to that of LEA38 (Figure 4). Since the LEA2.5-GFP construct was targeted to mitochondria, this indicates that an alpha-helix displaying a hydrophobic face of five residues, with a negatively charged residue at the interface, and two positively charged residues (separated by three residues in the projection) at the opposite end of the hydrophobic face, are essential conditions for mitochondrial targeting.

### 2.3. MTS Cleavage Site Determination for LEA38

**Figure 5:**
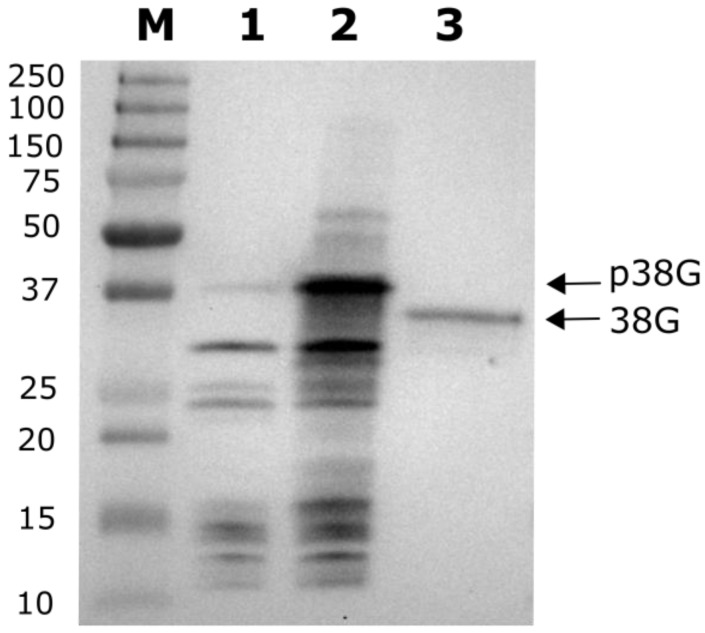
Comparison of the apparent molecular mass of the LEA38-GFP precursor and the mature LEA38-GFP. Precursors for LEA2-GFP and LEA38-GFP were synthesized in vitro. The mature LEA38-GFP (38G) was immunopurified from the leaves of an Arabidopsis overexpressor. Proteins were analyzed by Western blot using the anti-LEA38 antibody. This antibody was very specific to LEA38 and did not cross-react significantly with LEA2. 1, in vitro synthesized LEA2-GFP precursor (above 37 kDa); 2, in vitro synthesized LEA38-GFP precursor; 3, immunopurified mature LEA38-GFP. Arrows indicate the LEA38-GFP precursor (p38G) and mature LEA38-GFP (38G); M: molecular mass markers, with mass indicated in kDa.

Since mitochondrial transit peptides (30–50 residues) are often cleaved during the import process, in the case of LEA38, which has 97 amino acids, this could remove up to half of the precursor proteins, yielding a rather short mature protein. Another possibility is the absence of cleavage of the pre-sequence, which is often the case for mitochondrial membrane proteins [4]. The sub-mitochondrial localization of LEA38 has not yet been established, but its small size and the lack of predicted transmembrane helices (Table S1) do not favor a membrane localization. The existence of a cleavage site was questionable for LEA38, and we therefore attempted to determine the N-terminus of the protein using an Arabidopsis transgenic line constitutively expressing LEA38-GFP in mitochondria [25]. The LEA38-GFP was immunopurified from leaves and analysed by western blot using a specific anti-LEA38 antibody. The molecular mass of the protein isolated from leaves was compared with that of a recombinant LEA38-GFP synthetized in vitro (Figure 5). The recombinant LEA38-GFP precursor, produced by an *in-vitro* translation assay, displayed an apparent molecular mass of 39 kDa, consistent with its theoretical mass. LEA38-GFP which was purified from leaves exhibited a significantly lower apparent molecular mass (35 kDa), indicating that the protein was cleaved after mitochondrial import. The LEA2-GFP precursor synthesized in vitro was only slightly detected by the anti-LEA38 antibody, confirming its specificity (Figure 5).

Higher amounts of mature LEA38-GFP protein were immunopurified from leaf mitochondria of the overexpressing line, and the N-terminus was determined by Edman microsequencing. The results revealed that cleavage of the presequence occurred at two adjacent sites, after Y28 and A29, and thus upstream of the 11 amino acid segment (^33^AQGSVSSGGRS), which is specific to LEA38 (see Figure 1). The theoretical molecular mass of the mature LEA38GFP (35.7 kDa) is thus consistent with the western blot results (Figure 5).

### 2.4. Comparison of Experimental Data and In Silico Predictions

Six online subcellular location prediction programs (iPSORT, MITOPROTII, MitoFates, PProwler, Predotar, and TargetP1.1) were used to predict the location of LEA2 and LEA38 and related GFP constructs (Table 2). Most programs suggested mitochondrial localization (32 out of 42 combinations in Table 2) for the seven proteins, although four of these are empirically cytosolic. More of the programs predicted a mitochondrial localization for LEA2, which is cytosolic, than for the mitochondrial LEA38 (5 and 4, respectively). The predictions for the native LEA proteins and their translational GFP fusions are logically very similar, with only some slight differences in probabilities, and a single difference in the decision for TargetP1.1 (LEA2 chloroplastic/LEA2-GFP mitochondrial), but both had a rather low score (0.36). This program was the least accurate with this set of proteins since LEA2, LEA38, LEA38-GFP, and LEA2.5-GFP were predicted as chloroplastic proteins and LEA38del-GFP as a secreted protein. The overall comparison of predictions with experimental localizations, including the series of mutant proteins, confirms that none of the programs are sufficiently accurate to predict the subcellular localisation of polypeptides with confidence (Table 2). Three of the programs include the predicted length of pre-sequences in their analysis. As shown in Table 2, predicted MTS displayed various lengths (28 to 43 amino-acids), with cleavage sites upstream or downstream from the 11 amino acid sequence that is required for correct mitochondrial targeting of LEA38-GFP. The MitoFates program provided the best results with the prediction of the genuine cleavage site of LEA38 (after Y28).

## 3. Discussion

LEA2 and LEA38 are two paralogous proteins of the LEA_3 family with very similar primary sequences but different subcellular locations. The LEA_3 family is specific to higher plants, and both experimental data in Arabidopsis [25] and targeting predictions for 390 proteins of the family strongly suggest that mitochondrial localization is a characterizing feature of this family. Thus, it is reasonable to assume that LEA2 has lost its original mitochondrial localization. The primary objective of this study was to uncover the structural changes that occurred during evolution by identifying the key structural features of LEA38 that, when incorporated into LEA2 by mutation, are able to redirect the mutated LEA2 to mitochondria. We also expected this experimental model to provide original information about the import of mitochondrial proteins.

The main difference between LEA38 and LEA2 sequences is an eleven residue-long stretch of aminoacids (^33^AQGSVSSGGRS) which is specific to LEA38, and which was therefore a good candidate for a role in mitochondrial targeting. Indeed, its deletion abolished the mitochondrial localization of LEA38. However, its insertion in LEA2 had no effect on the subcellular targeting of the protein, which remained cytosolic; two additional mutations of LEA2 (Q11V and a charge inversion E17K/K18E) were required to redirect the protein to mitochondria. This confirmed that the ^33^AQGSVSSGGRS sequence was essential but not sufficient for mitochondrial targeting. Interestingly, we could determine that this stretch of amino acids was not included in the presequence, but located a few amino acids after the MTS cleavage site (occurring after residues 28 and 29). This sequence could therefore be involved in the recognition of the pre-sequence cleavage site by proteases. Its enrichment in Ser residues supports this hypothesis, since Ser residues were frequently found downstream of presequence cleavage sites which have been established for 62 Arabidopsis and 52 rice mitochondrial proteins [13].

In agreement with previous studies [17,34,35,36], we found that the distribution of charged and apolar residues in the N-terminus was critical for mitochondrial import, and the results further support the requirement of an amphiphilic alpha helix structure with positive charges in the MTS. The analysis of helical projections in the MTS of the native and mutant proteins allowed us to identify critical structural features for the mitochondrial import of LEA38. The hydrophobic face must comprise five residues (four is not enough) and the hydrophilic face must harbor two positively charged residues, separated by three residues. Thus, the positive charges are positioned at the opposite end of the hydrophobic-hydrophilic interfaces of the helix. Finally, the single negatively charged residue (Glu) must be adjacent to the hydrophobic face of the helix. In this context, the effect of the charge inversion (^17^EK to ^17^KE) which was required for mitochondrial targeting of the LEA2 mutant is well justified. It has a major effect on the helical projection, restoring the original charge distribution in LEA38 MTS. All these features are necessarily important for mitochondrial import. The amphiphilic helix with its positive charges constitutes a target for interaction with cytosolic and mitochondrial HSP70 chaperones, with the latter being involved in the translocation of precursors [4,11]. The hydrophobic side and the positively charged side of the amphiphilic alpha-helix might be recognized sequentially by distinct TOM receptors [17]. Eventually, positively charged residues in the MTS could contribute to the subsequent translocation across the inner membrane, which is driven by membrane potential [4].

Two adjacent cleavage sites of the MTS were determined by Edman sequencing in LEA38, at positions ^28^Y↓^29^A and ^29^A↓^30^A (arrow indicates cleavage). For the first cleavage site, the surrounding sequence {^24^F-R-R-G-Y↓A↓A-T-A} conforms well with the consensus motif {R-X-(F/Y/L)↓(S/A)-(S/A/T)-X} of the “-3R group” plant MTS [13]. In the processing of -3R group proteins, after cleavage by the MPP, the additional protease ICP55 (Intermediate Cleaving Peptidase of 55 kDa) cleaves the N-terminal amino acid from the intermediate first generated by MPP [37]. In the case of LEA38, we postulate that the presence of two successive Ala residues in the N-terminal sequence ^28^YAATA of the protein after cleavage by MPP lowers the peptidase specificity of ICP55, releasing two mature proteins, each with an Ala N-terminus. Our results also highlight the importance of residues beyond the MTS cleavage site since the LEA38del mutant, in which 11 residues were deleted from position 33, lost its mitochondrial location. Further mutation studies would be required to predict how many residues downstream of the cleavage site are essential.

**Figure 6:**
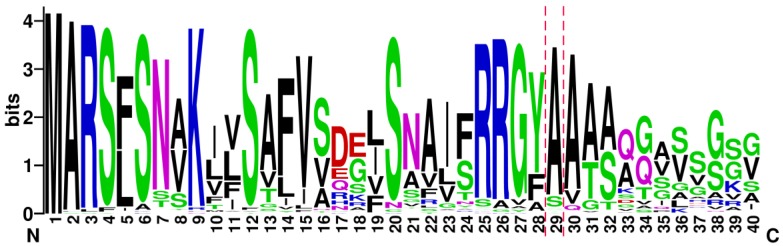
Sequence logo analysis of the N-terminal part of orthologs of LEA38. The first 40 amino-acid of 100 LEA_3 proteins sequences with the best scores in a BLASTP search were used to build the sequence logo. The mitochondrial targeting cleavage sites of LEA38 are indicated with the red dotted lines.

Since the vast majority of LEA_3 proteins were predicted to be mitochondrial, we built sequence logos using the first 40 amino acids of the 100 LEA_3 proteins with the best BLASTP score among the 390 sequences used for subcellular prediction analysis (Figure 6). The sequence logo illustrates the high sequence conservation in the N-terminal part of the proteins, and allows the establishment of a more precise consensus around the cleavage site {^25^R-R-G-(Y/F)**↓**A**↓**A-(A/T)-(A/S)} for the LEA_3 family. Interestingly, the corresponding sequence of LEA2 {^25^R-R-G-F-A-A-A-A-K-T} perfectly matches the consensus, and therefore the loss of mitochondrial location for the paralog of LEA38 is essentially due to a loss of targeting information, which does not affect the cleavage site motif. This appears logical since processing occurs within mitochondria during or upon import [7]. However, since LEA2-GFP expressed in protoplasts is apparently not cleaved [25], it also indicates that there is no protease activity in the cytosol with specificity equivalent to those of the mitochondrial processing peptidases. Limiting the processing peptidases to organellar locations should be crucial for the efficiency of import, and the high stability of LEA2-GFP in protoplasts supports the total lack of mitochondrial-like processing peptidase activity in the cytosol.

The coding sequences of LEA2 and LEA38 are very similar (71% identity), and both the length (96 vs. 100 nucleotides) and position (72 vs. 74) of the single intron are well conserved (Figure S1). The intron sequences are more divergent than the coding sequences, which reflects the lower evolutionary constraint on introns [38]. The high sequence similarity of sequences strongly supports the idea that LEA38 and LEA2 genes are paralogs resulting from one of the several whole genome duplication events in Arabidopsis [39]. The fact that all members of the LEA_3 family were predicted with high confidence to be mitochondrial strongly suggests that the ancestor of *LEA2* and *LEA38* genes encoded a mitochondrial protein, and that the protein encoded by the *LEA2* gene lost its mitochondrial localization after duplication. Gene duplication has been intensively reported as a very important evolution process leading to neofunctionalization (new function and/or expression pattern of one of the two duplicates) or subfunctionalization (division of ancestral functions and/or expression pattern between the two paralogs) [39]. Protein subcellular relocalization of duplicated genes has been observed in yeast [40], mammals [41], and plants [42]. Neolocalization (new localization for the copy product) or sublocalization (division of ancestral subcellular localizations between the paralogs) may contribute to the maintenance and functional divergence of genes pairs, and changes of expression patterns associated with protein relocalization were observed in Arabidopsis [42]. This is also the case for *LEA2* and *LEA38* genes that differ in their expression at the transcript level (data available at http://bar.utoronto.ca/efp2/Arabidopsis/Arabidopsis_eFPBrowser2.html). *LEA2* appears poorly expressed compared to *LEA38* in all developmental stages but its expression increases upon cold stress. *LEA38* is highly expressed within mature pollen, and in other tissues, its expression can be induced in response to osmotic stress and biotic stress. We can conclude that evolution of the paralogs led to a cytosolic sublocalization of LEA2, likely by the loss of mitochondrial targeting resulting from a few mutations, according to our experiments. Whether the neolocalization of LEA2 paralogs was associated with a new function for LEA2 in the cytosol is a difficult question to answer, because there is little information about the role of LEA_3 proteins. LEA38 (referred as AtLEA5) was shown earlier to complement an oxidant-sensitive yeast mutant, and its overexpression of *AtLEA5* in Arabidopsis increased oxidative stress tolerance [31]. In another study using over-expressor and anti-sense lines, LEA38 (referred as SAG21) was shown to interfere with leaf senescence, root development, and pathogen defense, which led to the proposal that LEA38 was involved in ROS signaling [32]. Interestingly, *SAG21* emerged from a genome-wide associated study as a major candidate gene for the local adaptation of Arabidopsis ecotypes [33]. We found that the discriminating single nucleotide polymorphism (SNP) of *SAG21* in this study was associated with an F/I substitution in position 24, in the MTS and just a few amino acids before the cleavage site (see Figure 6). It would be of interest in this context to determine if this substitution could have an effect on the import of LEA38. Still, the molecular function of LEA38 remains enigmatic, and to our knowledge, nothing is known about LEA2. If the latter performed a similar function as LEA38 in the cytosol, it would have to do so with an additional N-terminal extension (the part corresponding to LEA38 MTS) representing more than 30% of the length of mature LEA38 polypeptide. It must be recalled that, besides LEA38, two other very similar LEA_3 proteins (LEA37, LEA41) are also targeted to mitochondria [25], and therefore the loss of mitochondrial targeting for the *LEA2* gene product could possibly be compensated for by the other proteins. In conclusion, our results provide a rationale for the divergent subcellular localization of two LEA_3 protein paralogs with highly similar sequences, and suggest a scenario in which a few mutations resulted in the loss of mitochondrial targeting for one of the gene products, while the other one remained mitochondrial. We also provide arguments that support mitochondrial localization as a signature for LEA_3 proteins, and the first evidence, to our knowledge, that their MTS are cleaved upon import, releasing short mature proteins of around seven kDa. More research will be required to elucidate the molecular function of these small and intriguing plant mitochondrial proteins.

## 4. Materials and Methods

### 4.1. Plant Culture, Protoplast Isolation, and Transformation

*Arabidopsis thaliana* (Columbia-0 ecotype) wild type and transgenic lines were grown in potting compost Klassman 15 (Klasmann-Deilmann France SARL, Bourgoin-Jallieu, France) in a growth chamber (23 °C, 75% RH, 16 h light with an intensity of 100 μmol·m^−2^·s^−1^). Arabidopsis mesophyll protoplasts were isolated from a transgenic line expressing a mitochondrial mCherry protein and transformed according to the procedure described in [25].

### 4.2. Expression of Mutated Proteins

Synthetic genes encoding coding sequences of LEA2 (At1g02820) or LEA38 (At4g02380) with defined mutations indicated in Table 1 were obtained from GeneCust (Ellange, Luxembourg). LEA2, LEA38, and the mutated proteins coding sequence were cloned in the p2GWF7,0 vector from Plant System Biology (Ghent University, Ghent, Belgium) following the procedure described in [25]. These vectors were then introduced in Arabidopsis mesophyll protoplasts using polyethylene glycol mediated transformation [25].

### 4.3. Microscopy

The subcellular localition of fluorescent protein fusions in Arabidopsis mesophyll protoplasts were observed with a Nikon A1 laser scanning confocal microscope (Nikon France S.A, Champigny sur Marne, France). GFP, mCherry, and chlorophyll were excited with 488, 561, and 638 nm laser lines, respectively, with an emission band of 500 to 550 nm for GFP, 570 to 620 nm for mCherry, and 662 to 737 nm for chlorophyll autofluorescence.

### 4.4. In Vitro Production of Recombinant Proteins

LEA2-GFP and LEA38-GFP full-length proteins were obtained by coupled transcription-translation in vitro using the PURExpress kit (NEBS, Ipswich, MA, USA) using the recommended protocol.

### 4.5. Crude Mitochondria Isolation, and N-Terminus Sequencing

Mitochondria were isolated from rosette leaves of four week-old transgenic Arabidopsis plants expressing LEA38-GFP in mitochondria [25]. Leaves were grinded with a Waring blender in 10 mL isolation buffer (30 mM MOPS pH 7.8, 330 mM sorbitol, 2 mM EDTA, 1.5% *w*/*v* BSA) and then filtrated through eight layers of Miracloth. Chloroplasts and nuclei were removed by centrifugation at 1200× *g* for 45 s. The supernatant was further centrifuged at 5000× *g* for 5 min to obtain a crude mitochondrial fraction. GFP-tagged proteins were purified from crude mitochondria with the µMACS Epitope Tag Protein Isolation Kit (Miltenyi Biotec, Bergisch Gladbach, Germany) using the standard elution protocol. After separation by SDS-PAGE and transfer to the PVDF membrane, protein bands visualized by Coomassie Blue staining were excised for Edman microsequencing [43], which was performed by Proteome Factory AG (Berlin, Germany).

### 4.6. Protein Analysis by Western Blot

Proteins were separated by SDS PAGE and protein blotting was performed on PVDF membranes (Immobilon PSQ 0.2 µm, Merck KGaA, Darmstadt, Germany) using a transblot apparatus (Bio-Rad, Marnes-La-Coquette, France) and 10 mM CAPS pH 11, 10% (*v*/*v*) methanol as a transfer buffer [43]. The anti-LEA38 antibody (Genscript Biotech, Piscataway, NY, USA) was raised again the peptide (KKKGVEESTQKI) and used as a primary antibody at a dilution of 1:1000. As a secondary antibody, we used an anti-rabbit IgG coupled to horseradish peroxidase (Merck KGaA, Darmstadt, Germany) at a dilution of 1:50,000. The immunodetection was performed by incubating membranes in the Clarity^TM^ Western ECL reagent (Bio-Rad, Marnes-La-Coquette, France) and the emitted chemiluminescence was recorded by a Chemidoc Imager (Bio-Rad). Molecular masses were estimated using Precision Plus Protein Dual Color Standards (Bio-Rad).

### 4.7. Bioinformatics

Sequence alignments were performed with the LALIGN software (http://www.ebi.ac.uk/Tools/psa/lalign/) [44], Clustal Omega software (http://www.ebi.ac.uk/Tools/msa/clustalo/) [45], and EMBOSS Needle (https://www.ebi.ac.uk/Tools/psa/emboss_needle/nucleotide.html) [46]. Search for orthologs of LEA38 within the NCBI Reference Sequence protein database was performed with BLASTP 2.8.0 (https://blast.ncbi.nlm.nih.gov/Blast.cgi) using BLOSUM62 matrix and default parameters [47]. The HMMER software (https://www.ebi.ac.uk/Tools/hmmer/) was used to search for Pfam matches [48]. Subcellular localisations and pre-sequence cleavage site predictions were performed using IPSORT (http://psort.hgc.jp/form.html) [49], MITOPROT II (http://ihg.gsf.de/ihg/mitoprot.html) [50], TargetP 1.1 (http://www.cbs.dtu.dk/services/TargetP/) [51], Predotar (https://urgi.versailles.inra.fr/predotar/) [52], PProwler (http://bioinf.scmb.uq.edu.au:8080/pprowler_webapp_1-2/) [53], and MitoFates (http://mitf.cbrc.jp/MitoFates/cgi-bin/top.cgi) [54] software with default settings. Molecular weight and theoretical pI were calculated using ProMoST (http://proteomics.mcw.edu/promost.html) [55]. Secondary structure predictions were performed with Jpred4 (http://www.compbio.dundee.ac.uk/jpred/) [56]. Alpha-helix projections were obtained with the HeliQuest Web server (http://heliquest.ipmc.cnrs.fr/) [57], using the Analysis module with default parameters (helix type set to alpha) and a window size set to FULL.

## Figures and Tables

**Table 1:** Description of the different mutated protein constructs.

Mutation	Description
LEA2del	LEA2 without residues 33 to 37 (KTALD)
LEA38del	LEA38 without residues 33 to 43 (AQGSVSSGGRS)
LEA2.1	LEA38 (residues 1 to 43) + LEA2 (residues 38 to 91)
LEA2.2	LEA2 with (KTALD) replaced by (AQGSVSSGGRS)
LEA2.3	Same as LEA2.2 with Q11V mutation
LEA2.4	Same as LEA2.2 with inversion at positions 17 and 18 (EK into KE)
LEA2.5	Same as LEA2.2 with Q11V mutation and inversion at position 17 and 18

**Table 2:** Subcellular localisation and pre-sequence length predicted for LEA2, LEA38, and related mutated proteins. Six programs were used to predict the protein subcellular localisation of the indicated proteins (M, mitochondria; Ct, chloroplast; SP, secretory pathway). When available, probability is indicated in parentheses. The length of the predicted targeting sequence is indicated below each prediction for the three programs providing this analysis.

	IPsort	MitoFates	MITOPROT II v1.101	PProwler 1.2	Predotar	TargetP 1.1
**LEA2**	M	M (0.839)	M (0.958)	M (0.84)	M (0.43)	Ct (0.364)
	-	28	35	-	-	48
**LEA38**	Ct	M (0.544)	M (0.986)	M (0.90)	M (0.31)	Ct (0.493)
	-	28	43	-	-	46
**LEA2-GFP**	M	M (0.889)	M (0.949)	M (0.85)	M (0.43)	M (0.357)
	-	28	35	-	-	28
**LEA38-GFP**	Ct	M (0.672)	M (0.995)	M (0.85)	M (0.31)	Ct (0.429)
	-	28	43	-	-	46
**LEA38del-GFP**	Ct	M (0.760)	M (0.984)	M (0.92)	M (0.32)	SP (0.499)
	-	28	28	-	-	13
**LEA2.1-GFP**	Ct	M (0.493)	M (0.984)	M (0.88)	M (0.31)	Ct (0.539)
	-	28	43	-	-	54
**LEA2.5-GFP**	M	M (0.500)	M (0.924)	M (0.73)	M (0.35)	Ct (0.657)
	-	28	43	-	-	54

## Supplementary Materials

Supplementary materials can be found at http://www.mdpi.com/1422-0067/19/6/1620/s1.

## Abbreviations

MTS	Mitochondrial targeting sequence
GFP	Green fluorescent protein
LEA	Late Embryogenesis Abundant
