# Supplementary material for: Decoding the Divergent Subcellular Location of Two Highly Similar Paralogous LEA Proteins
Source: Int J Mol Sci. 2018 May 31;19(6):1620. doi: 10.3390/ijms19061620 (PMC6032150; doi:10.3390/ijms19061620)
Supplement: Supplementary file 1 [file ijms-19-01620-s001.zip › Fig S1.pdf]

**A**

```
                 10        20        30        40        50        60
LEA2     ATGGCTCGTTCTCTCGCTAACGCTAAGATCCAATCCGTCTTCGGCTCCGAAAAGCTCTCG
LEA38    ATGGCTCGTTCTATCTCTAACGTTAAGATCGTATCTGCTTTCGTCTCTCGTGAACTCTCC
                 10        20        30        40        50        60

                 70        80        90            100            110
LEA2     AACGCCGTTTTCAGACGAGGGTTTGCCGCCGCAGC----CAAAACGGCGTTA-----GAT
LEA38    AATGCTATCTTCCGACGTGGTTATGCGGCCACGGCGGCGCAAGGGAGCGTTTCGAGCGGT
                 70        80        90       100       110       120

                          120       130       140       150       160
LEA2     GGAAGC---------GTTTCGACCGCGGAGATGAAGAAGAGAGCTGGGGAAGCTTCAAGC
LEA38    GGAAGAAGTGGAGCTGTTGCTTCGGCTGTGATGAAGAAGAAGGGAGTGGAAGAATCAACC
                130       140       150       160       170       180

              170       180       190       200       210       220
LEA2     GAGAAGGCACCATGGGTTCCAGATCCTAAAACCGGTTATTACAGACCGGAAACCGTTTCT
LEA38    CAGAAGATTTCTTGGGTTCCAGATCCCAAAACCGGTTATTACAGACCCGAAACCGGTTCC
                190       200       210       220       230       240

              230       240       250       260       270
LEA2     GAGGAGATTGACCCGGCTGAGCTACGAGCTATTCTCTTGAACAACAAGCAATAA
LEA38    AACGAGATTGACGCGGCTGAGCTACGAGCAGCTCTCTTGAACAACAAGCAGTGA
                250       260       270       280       290
```

**B**

```
LEA2  intron   1 --------------GTAACATCTG-TTTCTGAAACAAAAACGTTATGTAT 35
LEA38 intron   1 GGTAAGTCTCTATTGT----TCTGTTTTCT---------ACTTTA----- 32

LEA2  intron  36 TGCTCTG---TTTTCTCGTCG-----------CTTTAACAAAACGCTGAC 71
LEA38 intron  33 TGCTCTGTTTTTTTCTTGTAGTTATGCTCTGTTTTTTTCTAAACGGTGTC 82

LEA2  intron  72 GTTTTGGATTTTG-GATTCTCTTCAG 96
LEA38 intron  83 GTTTT---TTGTGTGAT----AACA- 100
```

**Supplementary data Fig. 1. Comparison of CDS (A) and intron (B) nucleotide sequences of LEA2 and LEA38 genes.** CDS alignments were performed with LALIGN Pairwise Sequence Alignment (Huang and Miller, 1991). Intron global alignments were done with EMBOSS Needle (Needleman and Wunsch, 1970). Default settings were used for both alignments. Identical nucleotides are indicated in red.
