# Supplementary material for: Decoding the Divergent Subcellular Location of Two Highly Similar Paralogous LEA Proteins
Source: Int J Mol Sci. 2018 May 31;19(6):1620. doi: 10.3390/ijms19061620 (PMC6032150; doi:10.3390/ijms19061620)
Supplement: Supplementary file 1 [file ijms-19-01620-s001.zip › Table S1.pdf]

Table S1.Transmembrane topology predictions for mature LEA38. The sequence of mature LEA38 was submitted to six programs designed for the detection of potential transmembrane domains.

**Program**	**Transmembrane domain**
**CCTOP** (http://cctop.enzim.ttk.mta.hu)	none
**DAS-TMfilter** (http://mendel.imp.ac.at/sat/DAS/DAS.html)	none
**Phobius** (http://phobius.sbc.su.se/index.html)	none
**Predictprotein** (https://www.predictprotein.org)	none
**TMHMM Server v. 2.**0 (http://www.cbs.dtu.dk/services/TMHMM-2.0/)	none
**TMpred** (https://embnet.vital-it.ch/software/TMPRED_form.html)	none
